# Multiple colic intussusceptions

**DOI:** 10.1002/ccr3.2181

**Published:** 2019-05-11

**Authors:** Stylianos Tzedakis, Meredith Flacs, Benjamin Besse, Diane Goere

**Affiliations:** ^1^ Department of Oncologic Digestive Surgery Gustave Roussy Institute Villejuif France; ^2^ Present address: Saint Louis Hospital – AP‐HP Paris France

**Keywords:** bowel obstruction, large bowel intussusception, multiple intussusceptions, secondary bowel malignancy

## Abstract

Adult large bowel intussusception represents a small minority of intestinal intussusceptions. Main causes involve primary or secondary bowel malignancies. Although multiple small intestine intussusceptions have already been reported, simultaneous large bowel intussusceptions have not been described in the literature so far.

## 1 QUESTION

A 63‐year‐old woman with a medical history of non‐small cell lung cancer with adrenal, and pancreatic metastases under chemotherapy treatment since 2 years, presented with an acute 24‐hour rectal bleeding. What is the cause and how is this condition treated?

## 2 ANSWER

**Figure 1:**
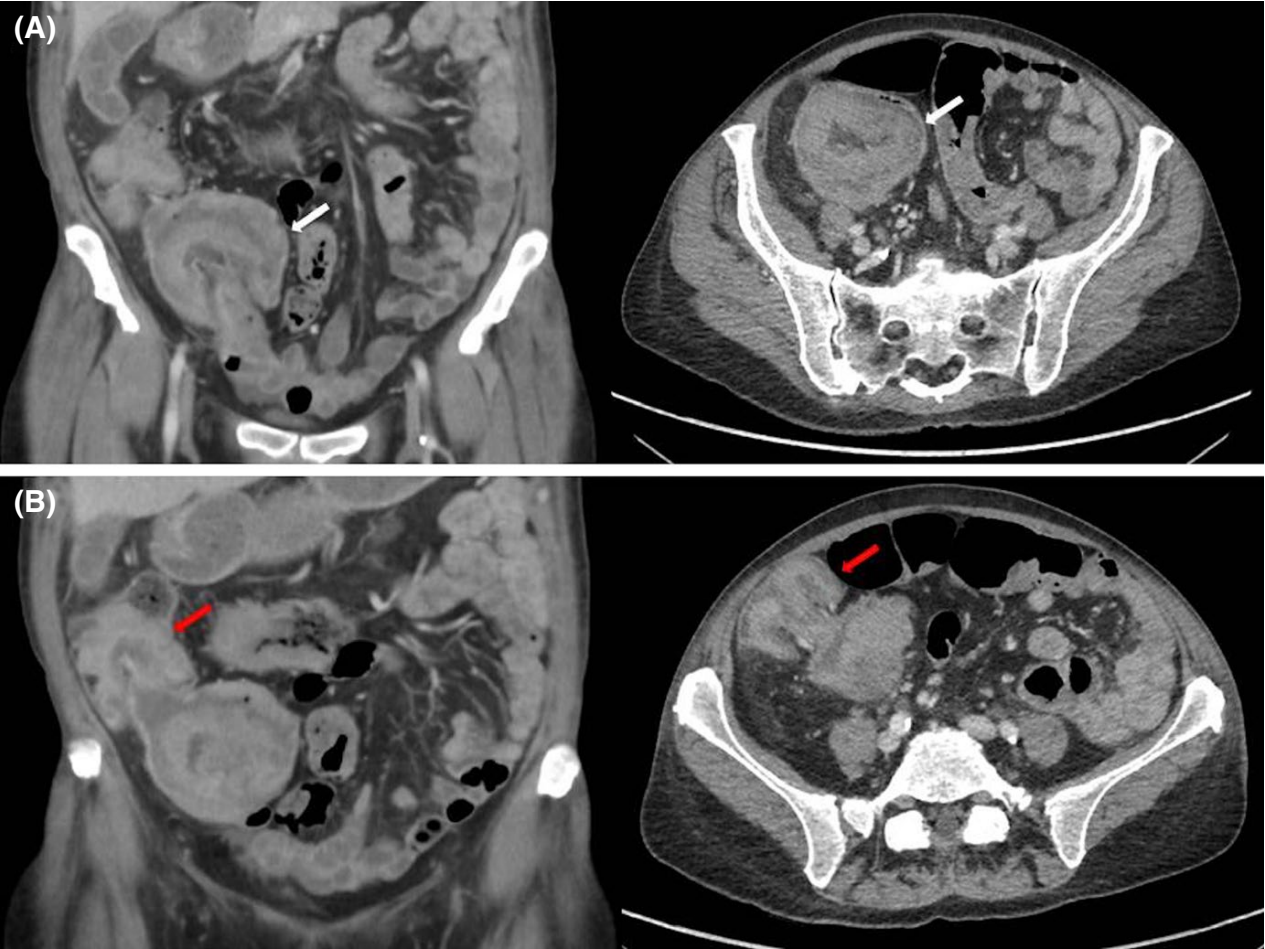
Coronal and axial planes of the computed tomography abdominal scan demonstrating a caeco‐colic (Panel A: white arrows) and a simultaneous colo‐colic intussusception (Panel B: red arrows) related to bowel metastasis of a non‐small cell lung cancer

This patient presented multiple colic intussusceptions. Recent colonoscopy had discovered multiple bronchogenic adenocarcinoma bowel metastases. Computed tomographic abdominal scan (Figure 1) revealed a double caeco‐colic (Panel A: white arrows) and colo‐colic intussusception (Panel B: red arrows) with a 35 and 40 mm lead point lesions, respectively. Conservative management was decided due to absence of bowel obstruction and spontaneous stop of bleeding.

Adult intussusception represents only 1%‐5% of all bowel obstruction and 5% of all cases of intestinal intussusception with the minority of cases involving the large bowel.^1^ Main causes involve primary bowel malignancies (64% of cases) especially for patients over 60 years of age.^2^ To our knowledge, multiple small intestine intussusceptions have already been reported in the literature but no multiple simultaneous large bowel intussusceptions have been described so far. Most of the patients with large bowel intussusception present with symptoms of chronic abdominal pain, acute bowel obstruction (36%‐82%), or bleeding (18%‐29%) often requiring surgical treatment. Conventionally, conservative approach has been described for cases where the probability of malignancy and ischemia is low.^1^ However, like in our case, it could also be considered for carefully selected patients, usually not fit for surgery, who are closely followed‐up.

## CONFLICT OF INTEREST

The authors declare that they have no conflict of interests.

## AUTHOR CONTRIBUTION

ST: study conception, acquisition and analysis of the data, drafting of the manuscript; MF: study conception and drafting of the manuscript; BB: study conception and critical revision of the manuscript; DG: study conception, analysis of the data, and critical revision of the manuscript.

## INFORMED CONSENT

Informed consent was obtained from the patient.
